# Increase of alcohol based hand rub consumption in hospitals participating in the German surveillance system

**DOI:** 10.1186/1753-6561-5-S6-P118

**Published:** 2011-06-29

**Authors:** M Behnke, F Schwab, C Geffers, P Gastmeier, C Reichardt

**Affiliations:** 1 Institute of Hygiene and Environmental Medicine, University Medicine Berlin - Charite, Berlin, Germany

## Introduction / objectives

A new national surveillance system (HAND-KISS) of alcohol based hand rub consumption (AHC) in hospitals was implemented to the German national nosocomial infection surveillance system (KISS) in the year 2008. We analyzed the differences of AHC increase of hospitals over three years.

## Methods

All hospitals participating in HAND-KISS send annually unit based AHC data to the HAND-KISS module. HAND-KISS is calculating AHC in Milliliter per patient day (ml/PD) stratified by specialty and by intensive care units (ICU's) and Non-ICU's. Reference data and the AHC distribution of 129 hospitals that provided baseline data in 2007 and follow up data in 2008 and 2009 were calculated. We grouped AHC baseline data of 2007 in quartiles and tested changes of AHC over three years between these groups using the Kruskal-Wallies-test. The following settings were analyzed: whole hospitals, ICU's only and Non-ICU's.

## Results

129 hospitals including 1659 units have consequently provided AHC data for the years 2007 to 2009. The overall median AHC increase from 2007 to 2007 was 30.7% (p<0.005), 21.5% for Non-ICU’s (p<0.005) and 36.5% for ICU’s (p<0.005). We did find significant difference in AHC change between the defined quartiles analyzing ICU's only (p<0.001) in contrast to whole hospitals (p=0.21) and Non-ICU's (p=0.173).

## Conclusion

AHC is a surrogate parameter to characterize hand hygiene behaviour in different settings. ICU's starting at a low level of AHC achieved a significant higher increase of AHC. Overall, our results show that there is room for improvement in all analyzed settings, irrespective of level of baseline AHC.

## Disclosure of interest

None declared.

